# Ultrasound-guided cross-pin technique for paediatric supracondylar humerus fractures: minimizing iatrogenic ulnar nerve injury

**DOI:** 10.1186/s12891-025-09001-3

**Published:** 2025-08-16

**Authors:** Xing Wu, Xiaoliang Chen, Xiongtao Li, Teng Wang, Jun Li, Xiantao Shen

**Affiliations:** Department of Pediatric Orthopedic Surgery, Tongji Medical College, Wuhan Children’s Hospital (Wuhan Maternal and Child Healthcare Hospital), Huazhong University of Science and Technology, 100 Xianggang Road, Wuhan, 430016 China

**Keywords:** Ultrasound, Cross-pin technique, Supracondylar humerus fractures, Ulnar nerve injury

## Abstract

**Background:**

Previous ultrasound-guided cross-pin techniques, which employ 90° elbow flexion, have demonstrated effectiveness; however, they may be associated with an elevated risk of iatrogenic ulnar nerve subluxation or dislocation. The aim of this study was to evaluate the efficacy and safety of a modified ultrasound-guided cross-pin technique for reducing the risk of iatrogenic ulnar nerve injury in paediatric patients with supracondylar humerus fractures.

**Methods:**

This retrospective study was conducted from December 2017- October 2019 and included paediatric patients with displaced supracondylar humerus fractures. The modified ultrasound-guided cross-pin technique was utilized to identify the ulnar nerve and confirm the medial pin position during pin placement with elbow in extension. The primary outcome measure was the incidence of iatrogenic ulnar nerve injury.

**Results:**

A total of 145 patients (mean age 5.8 years) were enrolled. There were 103 children with Gartland type III fractures, 35 with type II fractures, and 7 with type IV fractures. The incidence of iatrogenic ulnar nerve injury was significantly reduced to 0%. A total of 12 cases had abnormal pin insertions, including 11 cases in which the proximity of the inserted medial pin to the ulnar nerve was close and 1 case in which the ulnar nerve was directly violated by the pin. The rate of pin misplacement was 8.3%. At the latest follow-up, all patients demonstrated excellent and good functional outcomes according to the Flynn criteria.

**Conclusion:**

The ultrasound-guided cross-pin technique may serve as a viable alternative for reducing the risk of iatrogenic ulnar nerve injury in paediatric patients with supracondylar humerus fractures.

**Supplementary Information:**

The online version contains supplementary material available at 10.1186/s12891-025-09001-3.

Supracondylar humeral fractures are the second most common fracture in children, accounting for approximately 60% of elbow fractures in children, with a peak incidence between 4 and 7 years of age [1, 2]. The choice of treatment, that can be successfully managed, ranges from conservative to surgical methods [3, 4]. According to the modified Garland classification [5], type IIb, III, and IV fractures are considered unstable and require surgical intervention. The preferred treatment of choice is closed reduction and percutaneous pinning fixation [6]. Complications include iatrogenic nerve injuries [7], cubitus varus [8], and pin tract infection.

The primary goal of percutaneous pinning fixation is to maintain optimal stability. The two predominant pinning techniques are retrograde lateral pinning and cross-pinning. Although cross-pinning offers potentially superior biomechanical stability, it carries a risk of iatrogenic ulnar nerve injury. The incidence of iatrogenic ulnar nerve injury has been reported to be 0–6% [4, 9, 10]. Despite its widespread use in practice, crossed-pin fixation is avoided whenever possible [11]. However, it remains favoured for certain types of fractures because of its enhanced biomechanical stability [12].

The ideal safe technique of medial pin placement for avoiding ulnar nerve injury during cross-pinning remains controversial, with options including blind palpation, ulnar nerve stimulation, ultrasound guidance, or medial mini-open techniques [13–20]. Ultrasound offers real-time guidance and is broadly available, easily accessible, more affordable, without radiation exposure [21]. Physicians with no prior experience in ultrasound and limited training can readily employ it as a primary diagnostic tool for accurately diagnosing pediatric elbow fractures [22]. However, its intraoperative application has been limited due to a lack of standardized training and differences in healthcare settings [23, 24]. Currently, ultrasound is primarily utilized for preoperative localization of the ulnar nerve, rather than intraoperative guidance [20]. Soldado [16] first report that intraoperative ultrasound guidance facilitates direct visualization of the ulnar nerve and enables dynamic assessment of the pin trajectory. However, this approach employs 90° elbow flexion, which may poses an increased risk of ulnar nerve subluxation and dislocation.

Given that the cross-pin technique is routinely used in our department, determining the safest method becomes imperative. Accurate localization of the ulnar nerve and the medial pin during surgery is crucial to avoid iatrogenic ulnar nerve injury. In this study, we present a modified ultrasound-guided cross-pin technique with elbow in extension to reduce the risk of iatrogenic ulnar nerve injury in paediatric patients with supracondylar humerus fractures.

## Methods

We conducted a retrospective review of the medical records of children with supracondylar humeral fractures who underwent cross-pin fixation between December 1, 2017, and October 1, 2019. The inclusion criteria consisted of supracondylar humeral fractures treated with cross-pin fixation, whereas the exclusion criteria included cases without cross-pin fixation, surgical treatment performed outside the hospital, lack of postoperative follow-up or the presence of multiple fractures in the same limb, and preoperative ulnar nerve injury. For eligible patients meeting the inclusion criteria, electronic medical records were retrospectively reviewed to collect data on sex, age, injury characteristics, neurovascular status before and after surgery, follow-up outcomes, and postoperative complications. All patients who received medial pin placement (cross-pin fixation) were treated using an ultrasound-assisted technique. Fracture types were classified according to Wilkins’ modified Gartland classification system [5]. Both operations were performed by two surgeons (WX and SXT) possessing expertise in paediatric musculoskeletal ultrasound imaging techniques.

### Surgical technique

Closed reduction is performed using the standard technique: gentle traction on the injured limb and internal and external valgus correction in the coronal plane, followed by correction in the axial plane. A pin is passed through the lateral capitellum to engage the distal cortex for adequate fracture stabilization. A second pin was inserted if there was lateral column instability after the placement of one lateral pin. A pin punctures the anterior skin to enter the medial epicondylar region while visualization via ultrasound guidance to confirm proper positioning before bicortical fixation with insertion completion is achieved. The surgeon determined the pin size on the basis of the patient’s age and weight: 1.5 mm for children under 8 years of age or weighing less than 25 kg, and 2 mm for children aged 8 years or older, or weighing 25 kg or more.

#### Ultrasound technique for ulnar nerve monitoring

**Fig. 1:**
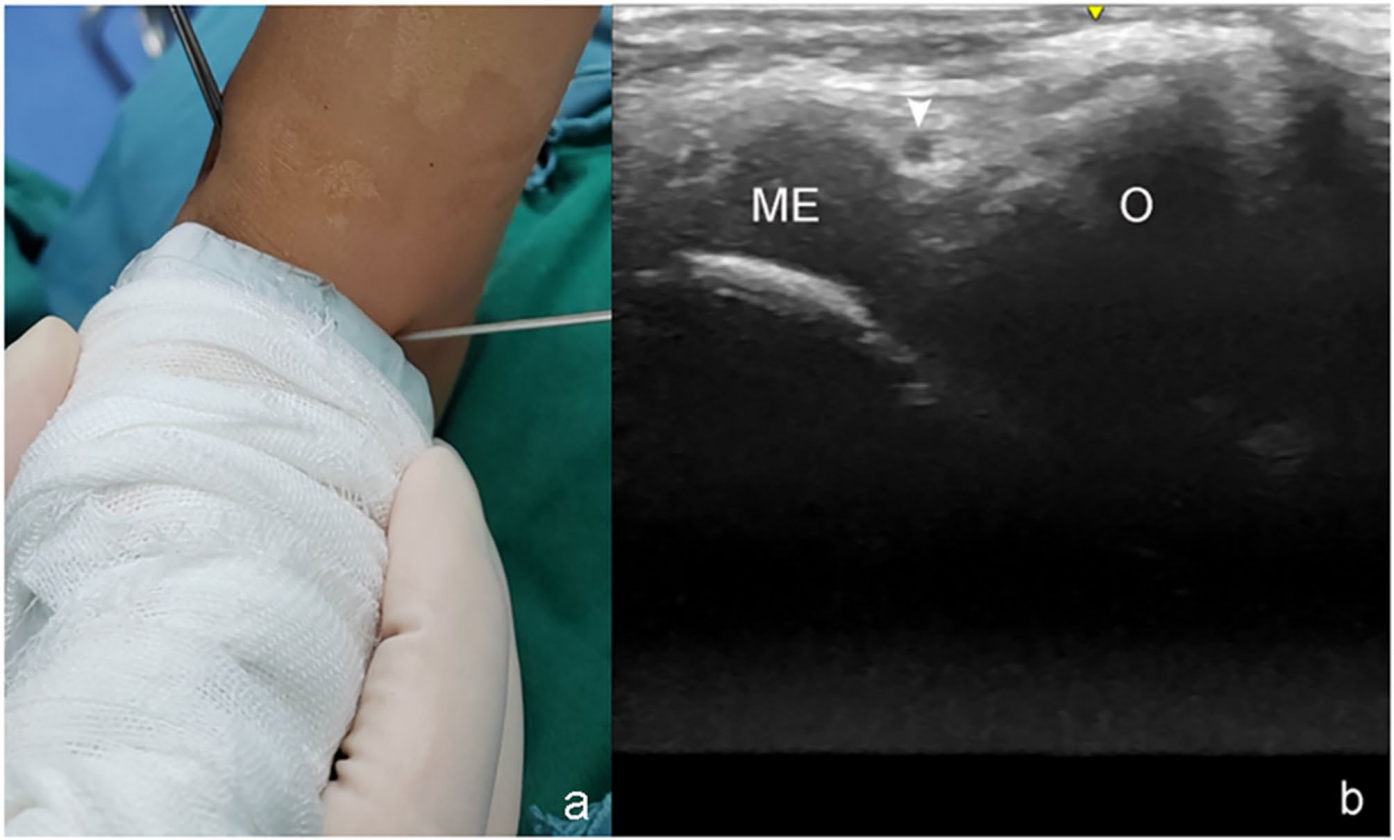
Photographs showing the positions of the transverse transducer (**a**), and corresponding ultrasound sections (**b**). The ultrasound reveals characteristic hypoechoic ulnar nerve (arrowhead), and the hypoechoic border of the medial epicondyle and olecranon with a typical acoustic shadow from the transverse sections. ME: Medial epicondyle of humerus; O: olecranon

A GE LOGIQ e ultrasound system (GE Healthcare, Milwaukee, WI, USA) equipped with a 7.0–12.5 MHz linear array transducer (GE Healthcare, Tokyo) was utilized for this purpose. During ultrasonographic examination, it is essential to initially ensure that an appropriate amount of physiological saline or alcohol is applied to both the transducer and the patient’s skin to optimize the propagation of ultrasonic waves. The positioning of the transducer was subsequently guided by alignment with reference to the medial epicondyle of the humerus for accurate ultrasound imaging. When transverse sectional images show the medial epicondyle of the humerus and the olecranon process of the ulna, the anatomical relationship between the ulnar nerve and the medial epicondyle can be ascertained before the pin placement (Fig. 1). In this plane, ulnar nerve subluxation or dislocation can be observed.

**Fig. 2:**
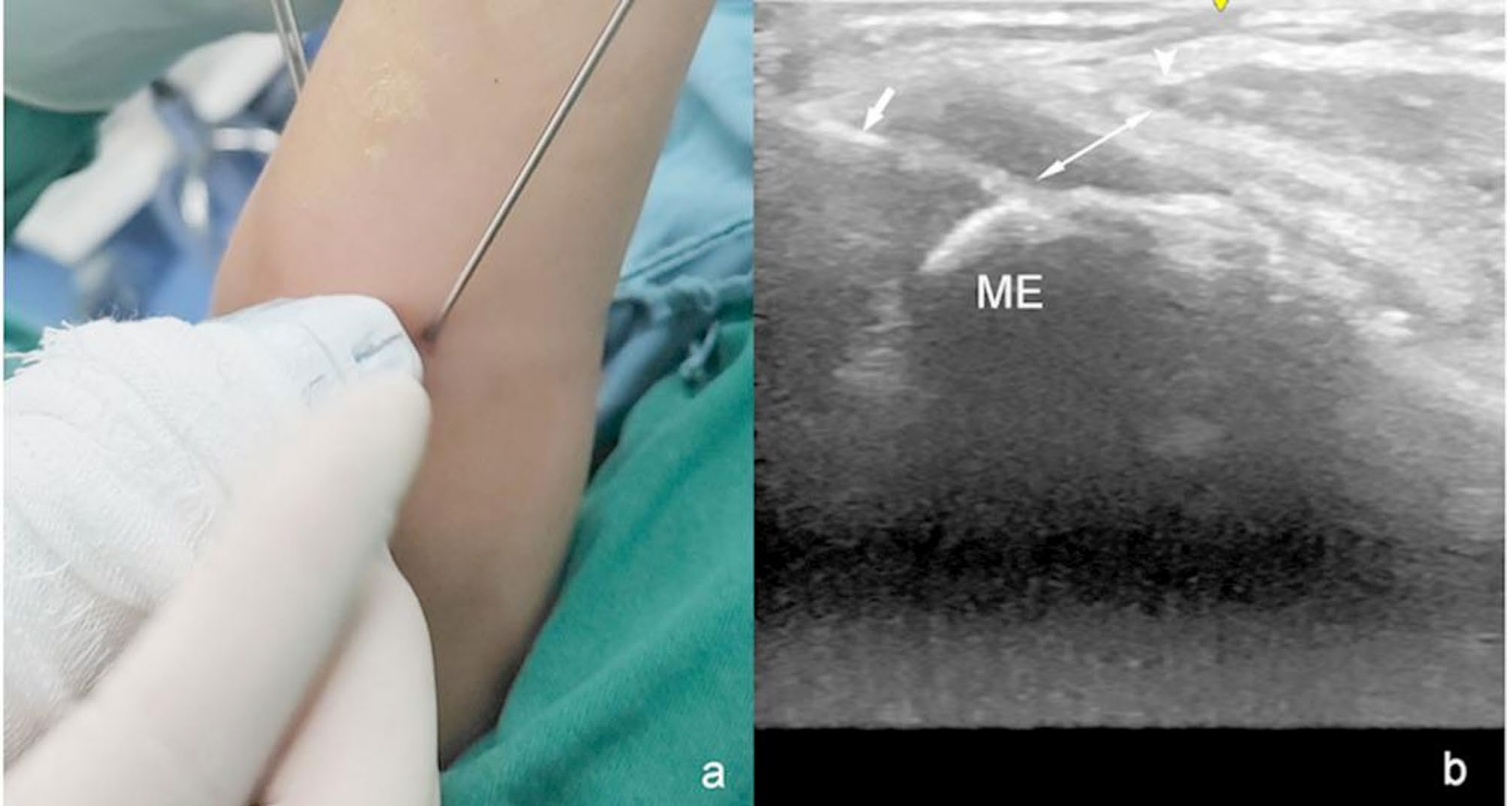
Photographs showing the positions of the oblique transducer (**a**), and corresponding ultrasound sections (**b**). Oblique section ultrasound in an elbow extension showing the relationship between the ulnar nerve (arrowhead) and medial pin (arrow) can all be seen. ME: medial epicondyle of humerus; double-ended arrow: the distance between ulnar nerve and medial pin

**Fig. 3:**
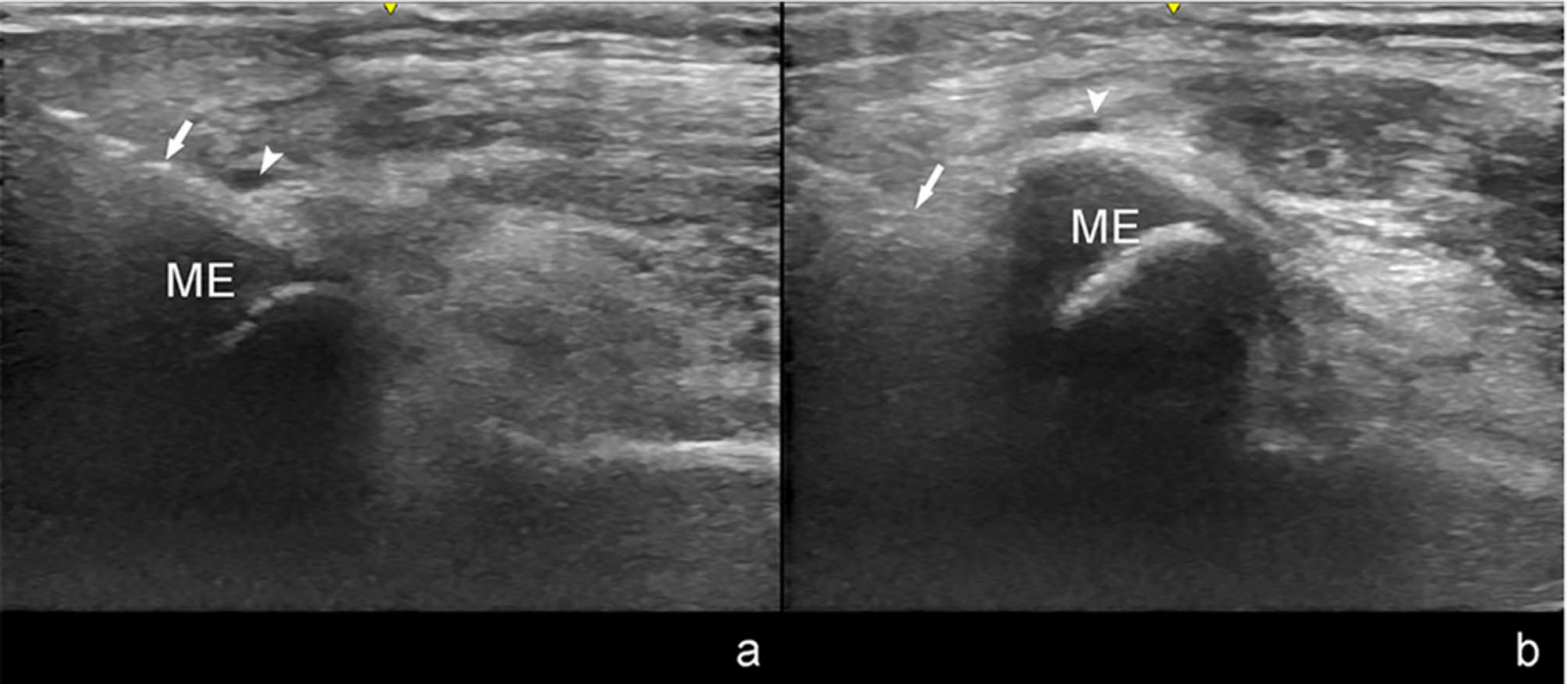
A 59-month-old boy, (**a**) Oblique section ultrasound showed the proximity of the inserted medial pin to the ulnar nerve. **b** The distance between the ulnar nerve and the medial pin could be seen after repeated pin insertion. ME: medial epicondyle of humerus; arrowhead: ulnar nerve; arrow: the medial pin

During the procedure, the assistant maintained elbow extension to prevent ulnar nerve instability, enhance visualization under ultrasound guidance, and stabilize the pin. The surgeon initially inserted the pin using freehand palpation, and then real-time visualization of the relationship between the ulnar nerve and the medial pin was achieved by rotating the transducer until it was parallel to the projection of the body surface of the pin. These oblique sections allowed visualization of the medial epicondyle as well as the pin and ulnar nerves simultaneously (Fig. 2). Dynamic observations of the ulnar nerve track were performed, and dynamic images were collected in Supplementary Video 1. When palpation was rendered challenging due to swelling, the pin was dynamically inserted in real-time under the guidance of an oblique ultrasound section to accurately reach the medial epicondyle. Once the safety of the pin insertion was confirmed, the surgeon advanced the pin further. After fracture fixation, ultrasound was again used to verify the localization of the nerve using oblique sections. When the proximity of the medial pin to the ulnar nerve or its residence within the cubital tunnel was of concern, the pin was immediately removed, and pin insertion was repeated to avoid ulnar nerve injury (Fig. 3).

Postoperation, neurologic examinations were closely assessed, and patients were followed up at our outpatient clinic. Clinical evaluation included assessment of the passive range of elbow movement and the difference in the “carrying angle” between the affected and unaffected upper limbs. In addition, postoperative evaluations included neurological and vascular examinations, as well as assessments for complications such as superficial infection, deep infection, and pin migration. The clinical results were graded according to the criteria of Flynn et al. [19], which are defined by motion loss in degrees and the loss of carrying angle in degrees. Iatrogenic nerve injury refers to any motor or sensory dysfunction within a specific nerve distribution that is not clearly documented prior to surgery. This comprehensive follow-up ensured that any potential complications were recorded.

## Results

A total of 145 patients with supracondylar humerus fractures were followed up and ultimately enrolled in this series. Table 1 presents demographic characteristics. The mean age of the children who underwent surgical fixation was 5.8 years (1.2–14.5 years). A total of 110 patients had modified Gartland type III or IV fractures, and the remaining 35 had type II fractures with either medial comminution or varus impaction. Preoperative nerve damage was recorded in 14 patients. These included 6 cases of anterior interosseous nerve injury and 8 cases of median nerve apraxia. All of the fractures were stabilized with 2 to 4 crossed K-wires. One lateral pin was used in 26 cases, and 2 lateral pins were used in 119 cases; 1 medial pin was used in 139 cases, and 2 medial pins were used in 6 cases. The medial epicondyle could be palpated in all patients. However, despite swelling, the ulnar nerve was not palpable in 48 of the 145 patients. Pins were removed at an average of 4.3 weeks (3.0-5.5 weeks). Postoperative casting took 5.1 weeks (3.0–6.0 weeks).

**Table 1:** Demographic characteristics

Number of patients	145
Age (years)	5.8 (range,1.2 ∼ 14.5)
Sex (boys: girls)	85/60
Laterality (right: left)	58/87
Fracture conffguration	
Modiffed Gartland type IIB with medial comminution	21
Modiffed Gartland type IIB with varus impaction	14
Modiffed Gartland type III	103
Modiffed Gartland type IV	7
Follow-up period (months)	13.4 (range, 11 ∼ 20)

Intraoperative ultrasound revealed a total of 12 cases of abnormal pin insertions, including 11 cases (7.6%) with the proximity of the inserted medial pin to the ulnar nerve and 1 case (0.7%) in which the ulnar nerve was considered to have been directly violated by the pin, which did not present with symptoms. The rate of pin misplacement was 8.3%. Prior to pin insertion, the ulnar nerve was nonpalpable in all 12 patients due to swelling, including 10 patients with modified Gartland type III fractures and 2 with modified Gartland type IV fractures. None of the 12 patients presented with neurological symptoms at the time of the operation.

Among the total number of patients, 145 patients were followed up for 11 to 20 months (mean 13.4 months). The clinical subgroup outcomes at the final follow-up are shown in Table 2. The fractures in all patients healed, and cubitus varus deformity was not observed. No cases of iatrogenic ulnar nerve injury, secondary fracture displacement, or compartment syndrome were observed. Postoperative complications included 1 case of radial nerve neuropraxia, which resolved spontaneously after 2 weeks. Two cases of pin migration were resolved after the removal of pins. Eleven cases of pin tract infection resolved after the removal of pins and oral antibiotics. One patient with a deep infection required an incision and drainage. At the final follow-up, clinical outcomes were routinely assessed using the Flynn criteria. No significant differences were observed between subgroups (*P* = 0.429). A total of 108 patients had excellent results, and 37 patients had good results, with an excellent and good response rate of 100%.

**Table 2:** Clinical outcomes

	Type II (*N* = 35)	Type III (*N* = 103)	Type IV(*N* = 7)
Flynn’s criteria			
Excellent *N*(%)	29	74	5
Good *N*(%)	6	29	2
Fair *N*(%)	0	0	0
Poor *N*(%)	0	0	0
Complications			
Iatrogenic radial nerve neuropraxia	0	0	1
pin migration	0	1	1
pin tract infection	2	9	0

A power analysis was conducted to assess the statistical capability of our study in detecting differences in ulnar nerve injury rates between our modified ultrasound-guided cross-pin technique and those reported in previous literature for alternative approaches. Existing evidence indicates an incidence of ulnar nerve injury of approximately 4.1% (20 out of 493 cases), whereas our technique demonstrated a 0% injury rate (0 out of 145 cases). Utilizing a one-sided t-test with a significance level of α = 0.05, the post-hoc power analysis revealed a power of 93.9%, suggesting that our study possesses sufficient power to demonstrate a significant difference in the risk of iatrogenic ulnar nerve injury when comparing our technique of medial pinning to blind palpation techniques. Conversely, based on current literature, the reported incidence of ulnar nerve injury is approximately 0.43% (3 out of 698 cases), and the post-hoc power analysis yielded a power of 3.1%. This finding suggests that there was no significant difference in the risk of iatrogenic ulnar nerve injury associated with our technique compared to medial mini-open techniques.

## Discussion

This study utilized a modified ultrasound-guided technique to evaluate the position of the ulnar nerve relative to medial pin placement. In our large single-centre case series of patients with supracondylar humerus fractures treated with cross-pinning fixation, we found no cases of iatrogenic ulnar nerve injury.

Conventional methods to avoid ulnar nerve injury during medial pin placement include blind palpation and manipulation of the nerve [19] and direct exposure of the ulnar nerve via the medial mini-open technique [13]. The incidence of ulnar nerve injury using blind palpation techniques has been documented to range from 0–11% [15, 25, 26]. In contrast, the mini-open technique, which allows for direct visualization at the pin entry site, has demonstrated a lower incidence of ulnar nerve injury, ranging from 0 to 0.43% [13, 14, 27, 28]. This direct visualization is considered more reliable than blind palpation [13, 14]. Although the scars from medial incisions are small, they remain conspicuous and may theoretically increase the risk of wound infection. A recent literature review reported that of 179 cases of iatrogenic ulnar nerve injury, 4 (2.2%) were treated with the mini-open technique, and 175 (97.8%) were treated with the blind palpation technique [7]. Nonetheless, Ercin [27] reported no statistically significant difference in the incidence of ulnar nerve injury between the medial mini-open and blind palpation techniques. Because ulnar nerve injury can occur at the time of pin placement [7], it is crucial to assess the relationship between the pin and the nerve immediately during pin placement to avoid potential injury. However, the presence of swelling and ulnar nerve subluxation significantly increases the risk of nerve injury when utilizing blind palpation methods. Our crossed-pin fixation technique also follows recent modifications to the blind palpation technique manoeuvre [15, 26]. Our findings demonstrate a favorable comparison with previous studies, as this technique effectively reduced the incidence of iatrogenic ulnar nerve injury to 0%. According to the post-hoc power analysis, this method significantly decreases the incidence of ulnar nerve injury compared to blind palpation, achieving a rate comparable to that of the medial mini-open technique [13].

Previous studies have demonstrated that instability of the ulnar nerve is a risk factor for potential complications associated with crossed pins [20, 29, 30]. Various modalities, including freehand palpation and ultrasound, have been employed to assess the stability of the ulnar nerve and to evaluate its pathways in the elbow tunnel [31, 32]. Ultrasound has been demonstrated to be an exceptionally safe and effective tool for visualizing soft tissue structures, enabling precise assessment of ulnar nerve stability and its pathways in the elbow tunnel [16, 20, 29, 32, 33]. However, previous studies have focused primarily on evaluating the ulnar nerve in healthy individuals or postoperatively, with limited descriptions of intraoperative ultrasound assessment [16, 20]. Similarly, the current study revealed that ultrasound offers good visualization of the ulnar nerve, rendering it a valuable tool for intraoperative assessment of its localization in patients with supracondylar humeral fractures. The utilization of ultrasound not only allows for accurate visualization but also facilitates early identification of the potential risk of nerve injury associated with the cross-pin technique.

Our technique enables monitoring of the relationship between the ulnar nerve and the medial pin while minimizing the risk of ulnar nerve injury. However, studies on the use of ultrasound to guide closed percutaneous placement are limited [16, 20]. Soldado [16] demonstrated that ultrasound can minimize the risk of iatrogenic ulnar nerve injury during the crossed-pin technique. However, their method involved detecting the ulnar nerve using coronal-sectional ultrasound with the elbow flexed at 90° to maintain visualization during pin insertion. Yet, directly assessing both pin placement and the ulnar nerve location in this section is challenging. Consequently, this technique indirectly assesses the nerve’s status by evaluating its mobility within the cubital tunnel. Furthermore, when the elbow is flexed to 90°, there is a potential risk of ulnar nerve instability. One study [20] reported that intraoperative ultrasound assessment could adjust the elbow extension range on the basis of stability evaluation of the ulnar nerve, thereby avoiding subluxation-induced neural injuries during pin insertion. Nonetheless, this method still cannot visualize both the pin and the ulnar nerve. In this study, a modified ultrasound monitoring technique was employed. Like prior studies [20, 29, 32], this study emphasizes the critical importance of performing pin insertion under elbow extension to prevent ulnar nerve subluxation. Preoperatively, an ultrasound assessment was performed to evaluate ulnar nerve subluxation. Intraoperatively, using a simplified oblique ultrasound plane with the elbow extended, the positional relationship between the pin and the ulnar nerve was precisely assessed. This approach simplified real-time ultrasound monitoring during pin insertion and facilitate better visualization. Moreover, this method enhances the feasibility and convenience of surgeons.

In the current study, the overall incidence of pin misplacement was determined to be 8.3%. The ulnar nerve was not palpable in 48 out of 145 patients, and swelling that obscured the medial epicondyle or ulnar nerve compromised the reliability of traditional medial pin insertion techniques. There was one instance (0.7%) of direct ulnar nerve penetration and 11 instances (7.6%) where the medial pin was positioned in close proximity to the ulnar nerve. Although these misplaced pins did not result in iatrogenic ulnar nerve injury, they potentially heightened the risk of significant injury. All patients necessitated intraoperative correction due to improper pin insertion. Similarly, Rasool [34] reported that early investigations into cases of ulnar nerve injury demonstrated that 33% were due to direct nerve penetration, while 50% resulted from nerve constriction by the cubital tunnel retinaculum. Although direct nerve penetration is relatively rare, the medial pin poses a risk of stimulating or entrapping the hypermobile ulnar nerve within the tunnel, potentially leading to nerve palsy [34, 35]. The proximity of the pin to the ulnar nerve is considered a potential risk factor; however, an exact safe distance between the pin and the ulnar nerve has not yet been established [30]. Our findings suggest that when ultrasound guidance indicated the pin was close to the ulnar nerve, immediate repositioning was performed to mitigate potential risks. Despite employing the mini-open technique, which allows for direct visualization, the risk of ulnar nerve injury cannot be eliminated. However, a study by Graff [7] reported that 91% of children with iatrogenic ulnar nerve palsies achieved complete recovery of nerve function at the final follow-up.

### Limitations

First, it is important to acknowledge that our single-centre case series has limitations in terms of generalizability. Therefore, future prospective studies are necessary to strengthen the conclusions. Additionally, the absence of iatrogenic ulnar nerve injuries in such a large sample strongly suggests the advantage of using this monitoring technique in the cross-pin technique. Second, ultrasound is operator dependent. However, in the present study, we utilized a standardized scanning protocol to obtain ultrasound images. Moreover, we still applied the previously described safe medial pin insertion technique in our study, which did not increase the difficulty of the surgical procedure. Third, in this study, paediatric orthopaedic surgeons conducted ultrasound examinations during surgery. Due to the limitations of retrospective studies, this study did not evaluate the learning curve. Proficiency in musculoskeletal ultrasound is crucial for the effective utilization of this technique. Concurrently, ultrasound imaging is gaining recognition among physicians for its effectiveness in diagnostic and interventional settings [22, 36–38]. The literatures where it were reported the operators with no prior ultrasound experience and only limited training is reassuring for clinicians beginning to use ultrasound diagnosticians. Musculoskeletal ultrasound courses in most studies were minimal, ranging from 1 to 4 h, concentrating on specific anatomical regions [37]. As a result, expertise in ultrasound imaging may become an essential competency for paediatric orthopaedic surgeons.

## Conclusion

This study represents the largest single-centre case series investigating the application of the ultrasound-assisted cross-pinning technique. The ultrasound-assisted technique is a safe, effective, and cost-efficient method for cross-pinning, with a low risk of iatrogenic ulnar nerve injury. Our technique can be used as an adjunct to other cross-pin methods. However, further randomized controlled trials are necessary to validate its efficacy, and adaptability across diverse healthcare environments, as well as training standardization, present significant challenges for surgeons.

## Supplementary Information

Supplementary Material 1.

Supplementary Material 2.

## Data Availability

The datasets used and analysed during the current study are available from the corresponding author on reasonable request.
