# Supplementary material for: Ultrasound-guided cross-pin technique for paediatric supracondylar humerus fractures: minimizing iatrogenic ulnar nerve injury
Source: BMC Musculoskelet Disord. 2025 Aug 16;26:792. doi: 10.1186/s12891-025-09001-3 (PMC12357387; doi:10.1186/s12891-025-09001-3)
Supplement: Supplementary file 1 — Supplementary Material 1. [file 12891_2025_9001_MOESM1_ESM.docx]

The procedure of the ultrasound is to obtain a dynamic image of the relationship between the ulnar nerve and the pin. Transducers obtain transverse sections of the humeral medial epicondyle and ulnar olecranon, clarifying the ulnar nerve's relation to the epicondyle. By gently rotating the transducer parallel to the pin, the ultrasound reveals the relationship between the ulnar nerve and the pin. ME: medial epicondyle of the humerus; arrowhead: ulnar nerve; arrow: placement of the pin is visualized.
